# CDK4/6 inhibitors rechallenge post-progression in HR-positive HER2-negative advanced/metastatic breast cancer patients: a meta-analysis of Kaplan–Meier-reconstructed individual-level data

**DOI:** 10.1186/s13058-026-02311-x

**Published:** 2026-05-27

**Authors:** Jasmina Veta Darkovski, Isabella Michelon, Gabriela Gazzoni, Isadora Mamede, Yujin Jeong, Ludimila Cavalcante

**Affiliations:** 1 Department of Obstetrics and Gynaecology, University Hospital Ulm, Prittwitzstr.43, 89075 Ulm, Germany; 2 Division of Hematology and Oncology, University of Virginia Comprehensive Cancer Center, 1240 Lee St, Charlottesville, VA 22903 USA; 3 Institute of Medical Assistance to the State Public Servant (IAMSPE), São Paulo, Brazil; 4 Department of Medicine, Federal University of São João del Rei, Divinópolis, Minas Gerais Brazil; 5 Icahn School of Medicine, Mount Sinai/Elmhurst, Elmhurst, NY USA

**Keywords:** Breast cancer, Endocrine therapy, Kaplan–Meier individual patient data meta-analysis

## Abstract

**Background:**

Cyclin-dependent kinase 4/6 inhibitors (CDK4/6i) combined with endocrine therapy (ET) are the standard of care for hormone receptor (HR)-positive, HER2-negative advanced/metastatic breast cancer (BC). However, the optimal strategy after progression remains uncertain.

**Methods:**

PubMed, Cochrane, and Embase databases were searched in April 2025 for phase II/III clinical trials evaluating CDK4/6i rechallenge in advanced breast cancer. We evaluated progression-free survival (PFS) and overall survival (OS) using Cox proportional hazards models, reporting hazard ratios (HRs) with 95% confidence intervals (CIs). Analyses were conducted on R (v.4.2.2).

**Results:**

Eight trials including 1396 patients were included, of whom 839 received CDK4/6i rechallenge plus ET, and 557 received ET alone. Median PFS was 5.8 months vs 3.7 months, with a 29% reduction in risk of progression or death (HR 0.71; 95% CI 0.63–0.81; *P* < 0.001). No OS benefit was observed (HR 1.04; 95% CI 0.70–1.55). Switching to a different CDK4/6i appears to confer greater benefit (HR 0.61; 95% CI 0.52–0.72), yet most patients who switched CDK4/6i received abemaciclib, which was associated with the longest PFS (7.9 months) compared with palbociclib (4.6 months) or ribociclib (5.5 months). Patients harboring ESR1 or PIK3CA alterations demonstrated a median PFS of 5.3 (3.6–7.4) and 4.7 (3.6–6.7) months, respectively.

**Conclusions:**

This meta-analysis suggests that CDK4/6 inhibitor rechallenge post-progression offers additional benefit to ET alone.

**Supplementary Information:**

The online version contains supplementary material available at 10.1186/s13058-026-02311-x.

## Background

Hormone receptor (HR)-positive human epidermal growth factor receptor 2 (HER2)-negative breast cancer (BC) remains the most prevalent subtype, accounting for roughly 70% of cases [1]. Historically, the mainstay of treatment for this population consisted of sequential hormone receptor-targeted therapy until exhaustion [2]. In recent years, the addition of cyclin-dependent kinase 4/6 inhibitors (CDK4/6i) has been shown to enhance antitumor activity and improve survival compared to endocrine therapy (ET) alone [3–6]. Thus, the combined regimen is now the preferred first-line therapy for patients with advanced HR-positive HER2-negative BC [7, 8].

Despite initial benefit, up to 50% of patients will eventually face disease progression within two years of a first-line CDK4/6i plus ET regimen [9, 10]. Currently, the choice of subsequent therapies is often based on genomic data (*i.e.,* PIK3CA, ESR1), toxicity, and patient preference. Some guidelines recommend switching targeted therapy agents or the ET backbone [11]. Nevertheless, the optimal treatment course following disease progression is yet to be established.

Evidence suggests that endocrine resistance stands as the primary driver of progression on CDK4/6i plus ET. If tumor cells remain sensitive to CDK4/6 inhibition, continuation of this regimen post-progression may potentially provide benefit. Accordingly, one of the treatment alternatives under investigation is rechallenge with CDK4/6i plus ET. Multiple clinical studies are currently investigating this approach in different scenarios: immediate rechallenge *versus* allowing for a drug-free interval; switching one component of the first-line regimen (ET backbone or CDK4/6i agent); and in patient subgroups defined by genomic alterations.

Results from these studies are mixed and still inconclusive. Findings from the randomized MAINTAIN and postMONARCH trials support this strategy [12, 13]. On the other hand, no meaningful progression-free survival (PFS) improvement was seen in two other randomized studies—PACE and PALMIRA [14, 15]. Prior studies and real-world cohorts have sought to address this question [16–20]. In this meta-analysis, we applied advanced methods of Kaplan–Meier-reconstructed individual-level data to pool survival outcomes of eight clinical trials including over 1000 advanced/metastatic HR-positive HER2-negative BC participants. We then comprehensively evaluated the benefit of rechallenge with CDK4/6i plus ET following progression on initial treatment in this patient population, including in a cohort harboring ESR1 and PIK3CA alterations.

## Methods

### Systematic review and protocol registration

This meta-analysis followed the Preferred Reporting Items for Systematic Reviews and Meta-Analyses (PRISMA) reporting guidelines [21]. The PRISMA checklist is available in Table S1. The study protocol was prospectively registered in the International Prospective Register of Systematic Reviews (PROSPERO; registration number [CRD420251041415]). This is a meta-analysis of previously published studies, and no human participants were recruited. Therefore, institutional review board approval and informed consent were not required.

### Databases and search strategy

We systematically searched PubMed, Embase, and Cochrane databases from inception to April 5, 2025, for studies that met our eligibility criteria. The search strategy combined controlled vocabulary (*e.g*., MeSH/Emtree terms) and free-text terms. The full search used in each database is detailed in Table S2.

### Eligibility criteria

We included English-language phase II/III randomized or single-arm clinical trials (CTs) exploring retreatment with FDA- or EMA-approved CDK4/6 inhibitors (palbociclib, ribociclib, or abemaciclib) plus ET following progression on prior CDK4/6i plus ET. Studies were included regardless of combination regimens and drug-free interval. Our exclusion criteria were: (1) abstracts from conference proceedings—abstracts were excluded to ensure inclusion of peer-reviewed studies with mature survival data and Kaplan–Meier curves required for individual patient data reconstruction. (2) retrospective data; (3) pooled analyses or other non-original studies; (4) case reports/series; (5) patient-reported outcomes reports; (6) CDK4/6i rechallenge following toxicity or for reasons other than progression; (7) studies reporting exclusively toxicity data, and (8) lacking survival data reported as Kaplan–Meier curves.

### Data collection and quality assessment

Authors independently screened studies (JVD and GG), selected reports for full assessment (JVD, GG, and IMi), collected data (JVD, IMi, GG, and IMa), and evaluated the quality of selected studies (IMa and YJ). For the latter, we used the Risk Of Bias In Non-randomized Studies of Interventions (ROBINS-I) [22], and Cochrane Risk-of-Bias tool for Randomized Trials (RoB 2) [23]. Disagreements at any stage, from screening to risk of bias assessment, were resolved by consensus or consulting other authors (IMi and LC).

### Outcomes and subgroups

Our main outcome of interest was overall PFS. Secondary outcomes were overall survival (OS), objective response rate (ORR), and clinical benefit rate (CBR).

We performed Kaplan–Meier-reconstructed individual-level PFS analyses as follows: (1) overall PFS of patients on CDK4/6i plus ET *vs* ET alone; (2) in patients using a different or the same CDK4/6i as in earlier lines; (3) according to the CDK4/6 inhibitor used in the rechallenge setting (ribociclib *vs* palbociclib *vs* abemaciclib); (4) by ET backbone in the rechallenge setting (Selective estrogen receptor modulator [SERM] vs selective estrogen receptor degrader [SERD] vs aromatase inhibitors [AI]); (5) according to mutational status (CDK4/6i plus ET rechallenge *vs* ET alone in patients with estrogen receptor 1 gene [ESR1] and phosphatidylinositol 3-kinase [PIK3CA] alterations). We also performed pairwise analyses to explore the impact of duration of 1st CDK4/6i; prior use of palbociclib and ribociclib in earlier settings; and visceral involvement in the PFS of patients under CDK4/6i plus ET rechallenge *vs* ET alone.

Some studies did not provide PFS Kaplan–Meier curves stratified specifically according to prior CDK4/6i exposure. Therefore, we pooled for analysis studies in which at least the majority (> 50%) of patients received a different CDK4/6i. Data considering the ET agent used in earlier lines was unavailable, thus, this analysis could not be carried out.

OS data were limited to only three studies, and we pooled it using Kaplan–Meier-reconstructed individual-level data therefore, no subgroups could be performed.

We used binary outcomes to explore the proportion of patients under rechallenge treatment achieving ORR and CBR but also carried out pairwise response rate analyses (CDK4/6i plus ET rechallenge *vs* ET alone).

### Sensitivity analyses

We performed sensitivity analyses by excluding non-randomized trials and studies on CDK4/6i plus ET combined with other agents from main PFS and OS analyses. The pooled effect size and recreated curves were then visually compared to our original analyses.

### Statistical analyses

As traditional meta-analysis methods are often limited to reporting time-to-event outcomes, we used the method described by Liu et al. for reconstructing individual patient data (IPD) from published Kaplan–Meier curves [24, 25]. Accordingly, we first extracted raw coordinates of time and survival probability of each group from the study Kaplan–Meier curves through the ‘getpoints’ function on R software. Then we reconstructed IPD using the ‘IPDfromKM’ R package. The accuracy assessment of reconstructed curves was performed by visual inspection and statistical measures (i.e., root mean square error, Kolmogorov–Smirnov test). Finally, we merged all IPD datasets to create a new dataset.

We applied Cox proportional hazards models to estimate the hazard ratio (HR) with 95% confidence intervals (CIs). We also estimated median survival time and 12- or 6-month PFS and OS for each group. Survival differences were explored via log-rank test. The proportional hazards assumption was assessed through the Grambsch-Therneau test [26]. Due to the small sample size of cohorts with ESR1 and PIK3CA mutations, we also used restricted mean survival time (RMST) as an alternative summary measure of the treatment effect, expressed as the mean difference in survival time over a 12-month follow-up period [27, 28].

For binary outcomes, analyses were conducted using either the number of events or the effect size (HR) and expressed as odds ratio (OR) or HR with 95% CIs. Proportional outcomes were reported in percentages. In case of studies with zero or extreme proportions, we applied double-arcsine and logit transformations, respectively. We applied random-effects models, and between-study heterogeneity was assessed using Cochran’s Q test and I2 statistics. Our threshold for significant heterogeneity was *p* < 0.10 and I^2^ ≥ 25%. Across all efficacy analyses, *p* values < 0.05 were deemed statistically significant. All analyses were conducted using R software (v4.2.2).

## Results

### Study selection

**Fig. 1:**
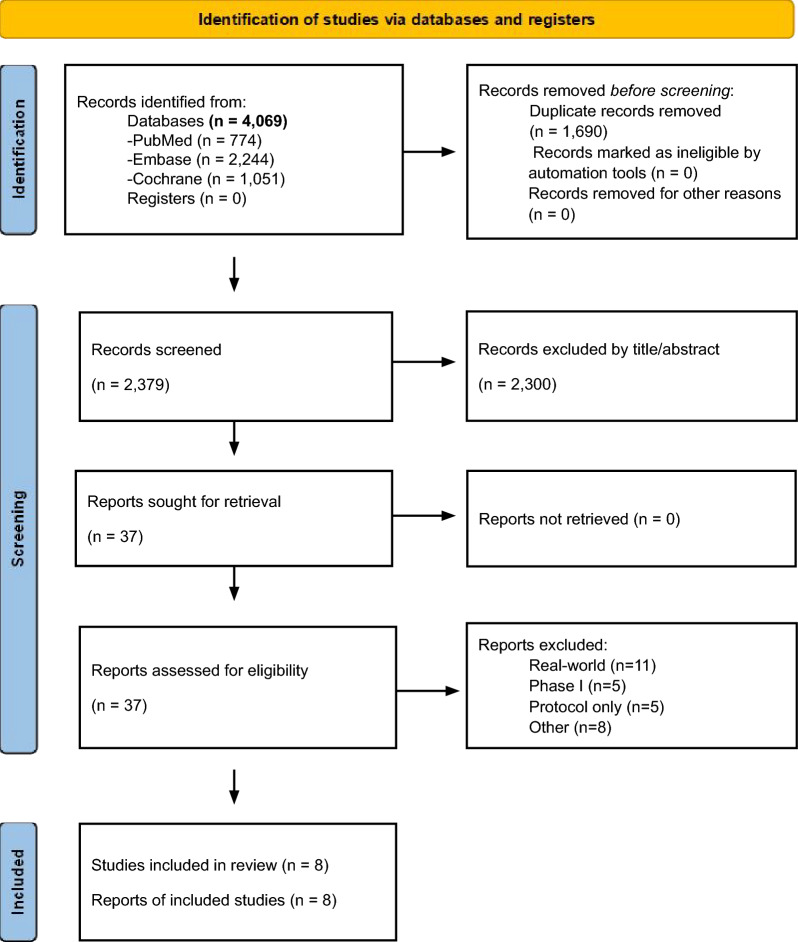
PRISMA 2020 flow diagram of study screening and selection. PRISMA: Preferred Reporting Items for Systematic Reviews and Meta-Analyses

Our search initially yielded 4,069 results, of which eight clinical trials (five randomized and three single-arm studies) were included (Fig. 1) [12–15, 29–32]. Reasons for exclusion of studies after full-text review are detailed in Table S3.

### Baseline characteristics

A total of 1396 patients were included in this meta-analysis, 839 among whom were rechallenged with CDK4/6i plus ET, and 557 received ET alone. Rechallenge was the second-line treatment in the advanced setting in most studies, and CDK4/6i of choice were abemaciclib (n = 350; 41.7%), palbociclib (n = 333; 39.7%), followed by ribociclib (n = 156; 18.6%). In all studies, ET used in the first line was switched to a different agent. Only in the study TRINITI-1, three out of 96 patients were treated with exemestane both in earlier lines and rechallenge setting. Fulvestrant was the most common ET backbone both in the CDK4/6i rechallenge (n = 534; 63.6%) and ET alone (n = 349; 62.7%) groups. Other baseline characteristics are detailed in Table 1.

**Table 1:** Baseline characteristics of studies included in this meta-analysis

Baseline characteristics	EMBER-3		MAINTAIN		PACE		PALMIRA		postMONARCH		BioPER	ELAINE 2	TRINITI-1
	CDK4/6i + ET	ET alone	CDK4/6i + ET	ET alone	CDK4/6i + ET	ET alone	CDK4/6i + ET	ET alone	CDK4/6i + ET	ET alone	CDK4/6i + ET	CDK4/6i + ET	CDK4/6i + ET
N of patients	139	195*	60	59	165	55	136	62	182	186	32	29	96
Treatment regimen	abema + imlu	imlu	ribo + F(N = 49)/ exe(N = 11)	F(N = 50)/ exe(N = 9)	palbo + F(N = 111) palbo + F + A(N = 54)	F	palbo + F(N = 120)/ LTZ(N = 16)	F(N = 58)/ LTZ(N = 4)	abema + F	F	palbo + F(N = 18)/LTZ (N = 9)/other(N = 5)	abema + LAS	ribo + exe + eve
Median age	NA	NA	55	59	palbo + F: 55 palbo + F + A: 58	58	59	61	58	61	59.5	60	58
Postmenopausal, N (%)	NA	NA	NA	NA	131 (79.4)	47 (85.5)	118 (86.6)	56 (90.3)	NA	NA	32(100)	25(86.2)	96 (100)
Visceral involvement, N (%)	NA	NA	36 (60)	35(59.3)	103 (62.4)	29 (52.7)	84(61.8)	37(59.7)	112 (61.5)	109 (58.6)	25 (78.1)	16 (55.2)	63(65.6)
Previous CDK4/6i used, N (%)													
Palbo	90 (64.7)	118 (60.5)	52 (86.7)	51 (86.4)	148 (89.7)	52 (94.5)	136 (100)	62 (100)	107 (58.8)	110 (59.1)	32 (100)	26 (89.7)	96 (100)
Ribo	37 (26.6)	56 (28.7)	6 (10)	8 (13.6)	9	1 (1.8)	0	0	61 (33.5)	61 (32.8)	0	2 (6.9)	12 (12.5)
Abema	10 (7.2)	19 (9.7)	2 (3.3)	0	7	2 (3.6)	0	0	14 (7.7)	14 (7.5)	0	4 (13.8)	0
Previous ET used, N (%)													
AI	139 (100)	195 (100)	49	50	NA	NA	120 (88.4)	58 (93.5)	182 (100)	186 (100)	18 (56.3)	28 (96.6)	89 (92.7)
SERD	–	–	11	9	NA	NA	16 (11.8)^‡^	4 (6.5)^‡^	–	–	14 (43.8)^‡^	23 (79.3)^‡^	37 (38.5)^‡^
Other	–	–	–	–	NA	NA	–	–	–	–	–	12 (41.4)^§^	–
Median duration of previous CDK4/6i, months	NA	NA	15.5	17	NA	NA	20.6	21.8	19	21	NA	NA	18
Prior CDK4/6i setting	neo/adjuv (n = 21) or 1L adv (n = 313)		adv (n = 119)		adjuv or adv (n = 169)		adv (n = 196)		adjuv (n = 3) or adv (n = 364)		adv	adv	adv
Intervening therapy allowed?	no		yes		yes		no		no		yes	yes	no
CDK4/6i + ET/ET-alone immediately post progression on prior CDK4/6i, N (%)	334 (100)		112 (94)		169 (76.8)		196 (99)		364 (98.9)		24 (75)	NA	96 (100)
Study design	Phase III RCT		Phase II RCT		Phase II RCT		Phase II RCT		Phase III RCT		Phase II	Phase II	Phase I/II**

*Abema* abemaciclib, *adj* adjuvant, *adv* advanced, *AI* aromatase inhibitor, *A* avelumab, *CDK4/6i* cyclin-dependent kinase 4/6 inhibitors, *ET* endocrine therapy, *eve* everolimus, *exe* exemestane, *F* fulvestrant, *imlu* imlunestrant, *LAS* lasofoxifene, *LTZ* letrozole, *Neo* neoadjuvant, *N* number of patients, *NA* not available, *palbo* palbociclib, *RCT* randomized clinical trial, *ribo* ribociclib, *SERD* Selective Estrogen Receptor Degrader

^‡^ fulvestrant; § tamoxifen; * only 140 patients were included in the survival analysis; ** we used findings reported for the cohort with antitumor and efficacy activity (n = 95)

### Main survival analyses

**Fig. 2:**
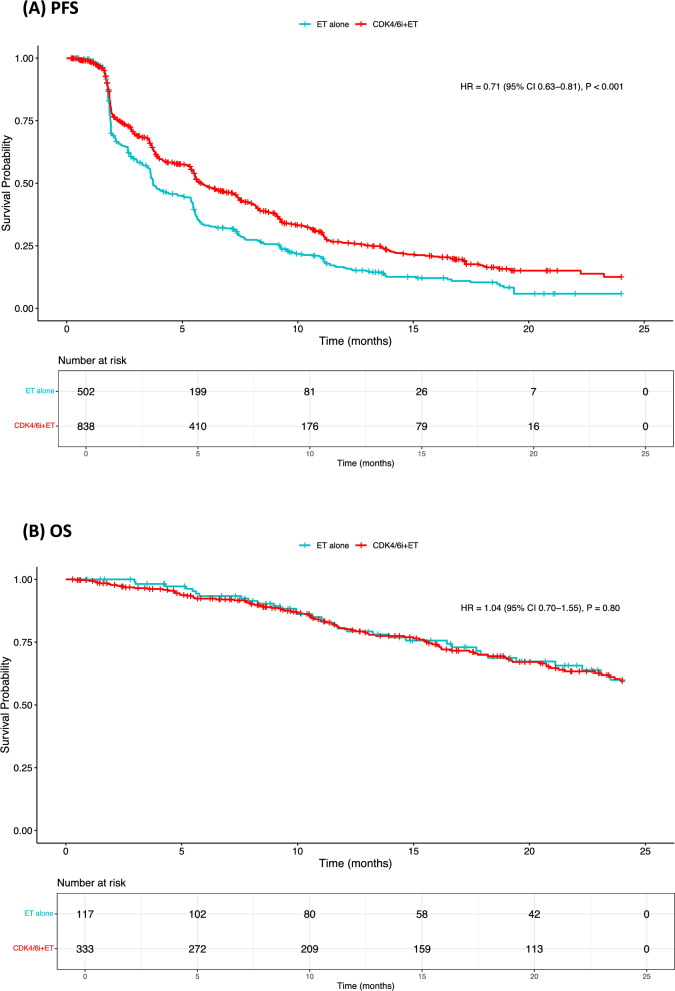
Progression-Free Survival (**A**) and Overall Survival (**B**) in CDK4/6i + ET rechallenge *versus* ET-alone. CDK4/6i: cyclin-dependent kinases 4/6 inhibitors; CI: confidence interval; ET: endocrine therapy; HR hazard ratio; PFS: progression-free survival; OS: overall survival

**Fig. 3:**
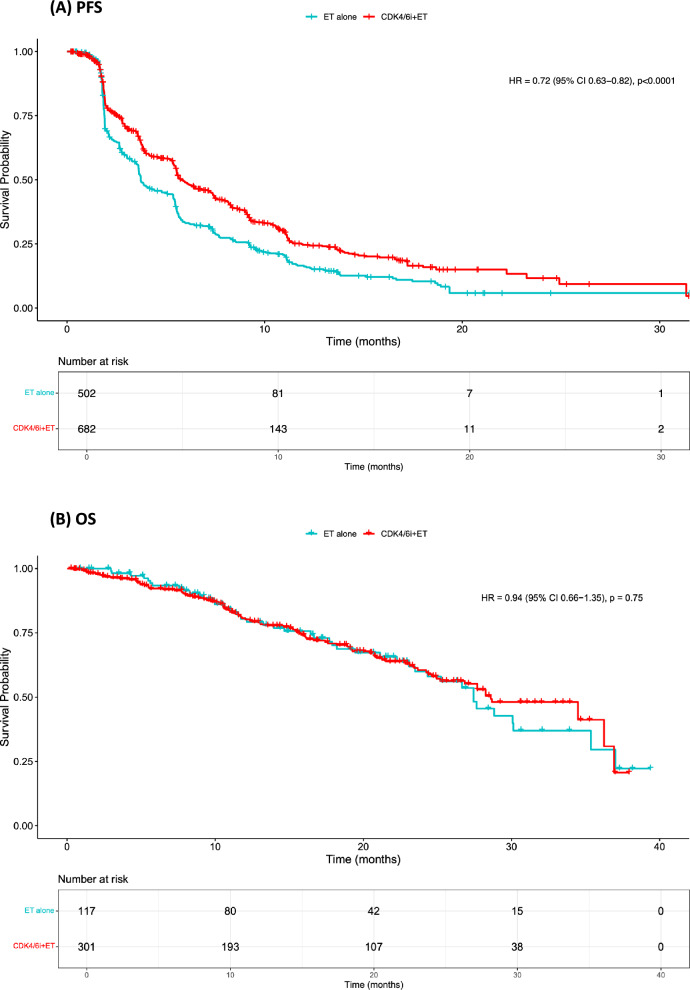
Progression-Free Survival (**A**) and Overall Survival (**B**) in CDK4/6i + ET *versus* ET-alone considering only randomized studies. CDK4/6i: cyclin-dependent kinases 4/6 inhibitors; CI: confidence interval; ET: endocrine therapy; HR hazard ratio; PFS: progression-free survival; OS: overall survival. Only the randomized studies PACE and PALMIRA had Kaplan–Meier curves for OS and therefore could be included in this analysis

In our primary analysis including 838 patients receiving CDK4/6i + ET and 502 receiving ET alone, median PFS was 5.8 and 3.7 months, respectively, with a 29% reduction in the risk of progression or death in favor of the CDK4/6i rechallenge arm (HR 0.71, 95% CI 0.63–0.81, *P* < 0.001). Fewer patients were included in the OS analysis and median survival was not reached for both groups (HR 1.04, 95% CI 0.70–1.55, *P* = 0.80) (Fig. 2). Analyses including only randomized studies revealed similar findings for both PFS and OS (Fig. 3).

### Subgroups

The PFS analysis of five studies with 497 patients who switched to a different CDK4/6i at rechallenge suggests a reduction in the risk of death or progression (HR for PFS: 0.61, 95% CI 0.52–0.72, *P* < 0.001) for this group compared to the cohort of 377 patients on ET alone (Fig. S1). This benefit was not observed among the 339 patients who continued the same CDK4/6 inhibitor used in earlier treatment lines compared with the 125 patients receiving ET alone (Fig. S1B). Median PFS was 7.3 months (95% CI 5.9–8.4) with a 12-month PFS of 32.9% (95% CI 28.5–38.1) in patients receiving a different CDK4/6i, compared with 4.6 months (95% CI 3.7–5.6) and 16.8% (95% CI 12.7–22.2) in those continuing the same CDK4/6i. Yet we highlight that most patients who were rechallenged with a different agent received abemaciclib upon progression. Therefore, it is unclear whether the observed benefit is attributable to this agent or the strategy of switching CDK4/6i.

Median PFS in months per CDK4/6i agent used in the rechallenge was as follows (Fig. S2): 4.6 (palbociclib), 5.5 (ribociclib), and 7.9 (abemaciclib). No direct comparisons between the three were made due to important heterogeneity factors (e.g., differences in ET arm, patient populations and treatment settings) that could affect the interpretability of data. The analysis of CDK4/6i versus ET stratified by CDK4/6i agent revealed that palbociclib was the only one that failed to show survival benefit over ET alone (Fig. S3).

The PFS analysis by ET backbone was also limited by a heterogeneous number of patients in each regimen: 535 on SERD, 106 on AI, and only 29 on SERM (Fig. S4). Median PFS was as follows: 6 months (95% CI 5.6–7.6) for SERD, 5.8 months (95% CI 3.7–9.7) for AI, and 14 months (95% CI 8.0-not reached) for SERM.

Other variables did not appear to play a role in the PFS (Fig. S5). Duration of 1st CDK4/6i > 12 months favored the rechallenge group, but the association was weak (HR 0.77, 95% CI 0.60–0.98, *P* = 0.04).

**Fig. 4:**
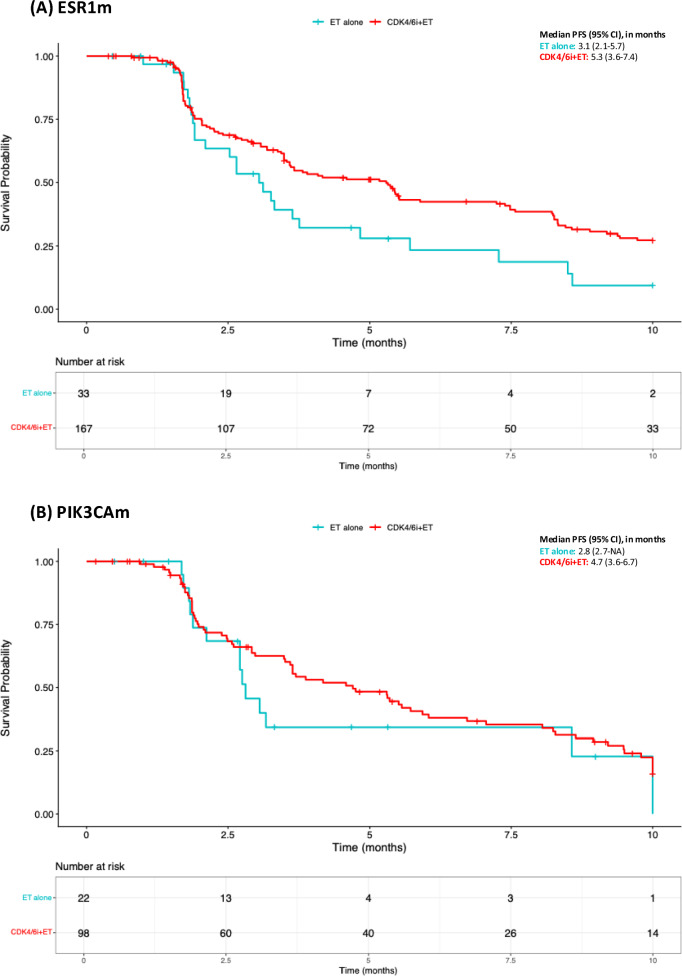
Progression-Free Survival in CDK4/6i + ET Rechallenge per Mutational Status: ESR1m (**A**) and PIK3CAm (**B**). CDK4/6i: cyclin-dependent kinases 4/6 inhibitors; CI: confidence interval; ESR1m: estrogen receptor gene mutated; ET: endocrine therapy; HR hazard ratio; NA: not available; PFS: progression-free survival

Limited data was available for patients harboring ESR1 and PIK3CA mutations (Fig. 4A). Therefore, findings should be seen as exploratory only and interpreted cautiously. Survival information was available for 200 patients harboring ESR1 mutations, of whom 167 received CDK4/6i plus ET and 33 were on ET alone (Fig. 4A). Median PFS was 5.3 months for the first and 3.1 months for the latter (*P* = 0.02), and PFS at 6 months was 42.3% and 23.4%, respectively. RMST analysis revealed a difference of 1.8 months in favor of CDK4/6i plus ET (Table S4, *P* = 0.01). PIK3CA mutations were detected in 98 patients treated with CDK4/6i plus ET (median PFS of 4.7 months, 95% CI 3.6–6.7) and 22 patients on ET alone (median PFS of 2.8 months, 95% CI 2.7-not reached; *P* = 0.28, Fig. 4B). The 6-month PFS was 39.3% for the CDK4/6i-combined arm and 34.2% for the ET alone. No significant RMST differences were observed between the groups (Table S4).

Median survival time and 12-month survival probability across all subanalyses are shown in Table S5. Results of the Grambsch-Therneau test for all analyses are also available (Table S6).

### Objective response and clinical benefit

Pooled ORR and CBR were 13.3% (95% CI 6.9–24.4%) and 38.9% (95% CI 32.0–46.2%), respectively, for the CDK4/6i plus ET cohort (Fig. S5). Borderline but nonsignificantly higher ORR was observed for CDK4/6i plus ET versus ET alone (OR: 2.1, 95% CI 1–4.5, *P* = 0.052, Fig. S6). The combination group achieved remarkably higher CBR (OR: 2.0, 95% CI 1.4–2.9, *P* < 0.001, Fig. S7).

### Sensitivity analyses and bias assessment

Sensitivity analyses yielded results consistent with the primary analysis, supporting the robustness of the observed PFS benefit of CDK4/6i rechallenge (Table S7). The three single-arm studies were judged at moderate risk, as all non-randomized studies are subject to bias due to confounding factors. Three randomized studies were judged as ‘some concerns’, as they were open-label investigator-assessed studies, which can introduce detection bias. The two other randomized studies met all prespecified criteria and were considered at low risk of bias (Table S8).

## Discussion

This Kaplan–Meier-reconstructed individual-level data meta-analysis included 1396 HR-positive HER2-negative advanced/metastatic BC patients, of whom 839 were rechallenged with CDK4/6i plus ET and 557 were on ET alone. We found a 29% reduction in the risk of death or progression (HR 0.71, 95% CI 0.63–0.81, *P* < 0.001) in favor of the rechallenge arm compared to ET alone. No OS benefit was seen, although OS data was immature across most trials. Among CDK4/6i used in the rechallenge setting, abemaciclib had numerically longer median PFS (7.9 months) compared to palbociclib (4.6 months) and ribociclib (5.5 months). Patients receiving a different CDK4/6i in the rechallenge appeared to derive greater benefit. Nevertheless, most patients receiving a different CDK4/6i agent were rechallenged with abemaciclib. Thus, the apparent benefit of this analysis may only reflect the superior activity of abemaciclib rather than the effect of switching CDK4/6i. Patients on CDK4/6i + ET rechallenge harboring ESR1 and PIK3CA mutations achieved survival rates comparable to those seen in our overall PFS analysis. Median PFS was 5.3 and 4.7, respectively.

Underlying resistance mechanisms to initial CDK4/6i plus ET treatment are key to supporting rechallenge strategies [29, 33]. Such mechanisms are complex and may be categorized into cell cycle-specific (*i.e.,* loss of RB, E2F, and CDK amplifications) and non-specific (*i.e.,* PI3K/AKT/mTOR signaling pathway) [34, 35]. Ultimately, they culminate in downregulation of hormone receptor expression and/or activation of alternative pathways. Tumor profiling analyses from patients progressing on first-line CDK4/6i plus ET indicate that endocrine-related resistance mechanisms were predominant over CDK4/6i resistance alterations. Thus, this provides the rationale for continuation of CDK4/6i inhibition while switching ET backbone.

In this meta-analysis, nearly all patients were treated with a different ET partner upon rechallenge. The overall analysis showed a longer PFS for those receiving the rechallenge strategy compared to ET alone. Yet it is unclear whether the benefit observed was related to switching to another CDK4/6i or to the specific agent used in the rechallenge setting. On one hand, two (PACE and PALMIRA) out of the five randomized studies included in our meta-analysis found no improvements in PFS compared to ET alone [14, 15]. Most patients (> 90% in PACE and all in PALMIRA) received palbociclib both in earlier lines and post-progression [14, 15]. The ET used in the rechallenge was fulvestrant, with only a small number of patients (n = 16) receiving letrozole in PALMIRA study. On the other hand, in MAINTAIN, postMONARCH and EMBER-3, a great percentage of patients also received palbociclib as first-line but changed CDK4/6i agents upon rechallenge [12, 13, 32]. The first explored ribociclib plus fulvestrant, whereas the two others investigated abemaciclib with fulvestrant or imlunestrant, respectively. Across all three studies, a PFS improvement superior to 25% was seen in favor of the rechallenge arm over ET alone.

Differences in activity of CDK4/6i agents warrant attention when interpreting these data. Abemaciclib has been associated with broader inhibitory activities compared to palbociclib and ribociclib [35]. Pre-clinical data demonstrate that abemaciclib targets kinases other than CDK4/6, such as CDK2/cyclin A/E, CDK1/cyclin B, some of which are also linked to treatment resistance [35]. Accordingly, we observed numerically longer PFS for the cohort rechallenged with abemaciclib. We highlight that abemaciclib was not commonly used in the first-line setting. Most patients who received this agent did so in the rechallenge setting only and fell under the ‘switching CDK4/6i’ category. Therefore, the apparent longer survival observed with the switching CDK4/6i strategy may have been driven by the use of abemaciclib. Moreover, we could not explore the effect of ET partners in this analysis due to an insufficient number of studies and patients. Yet, both postMONARCH and EMBER-3—which evaluated abemaciclib with different ET—reported remarkable benefit with CDK4/6i plus ET [12, 32].

Fulvestrant and AI agents were the most widely used ET backbones in our meta-analysis. Yet, several novel agents are emerging. Imlunestrant and elacestrant, next-generation oral SERDs, have shown encouraging activity in the phase III randomized EMBER-3 and EMERALD trials, respectively [32, 36]. Particularly, in ESR1m patients who previously received CDK4/6i plus ET, survival benefit was associated with SERDs monotherapy compared to standard ET [36]. In the EMBER-3 study, even better outcomes were seen when imlunestrant was combined with a CDK4/6i (HR 0.57, 95% CI 0.44–0.73, *P* < 0.001 in favor of imlunestrant plus abemaciclib over imlunestrant alone). ESR1 alterations lead to ligand-independent receptor activation and are one of the main drivers of endocrine resistance following AI-based regimens. Although single-agent elacestrant is the only ESR1m-specific targeted therapy approved, other agents such as the next-generation SERM lasofoxifene are also being investigated (ELAINE 2 and ELAINE III-NCT05696626) [30, 37].

Next-generation SERDs have thus become a preferred strategy for ESR1-mutated patients progressing on CDK4/6i. Our meta-analysis is consistent with EMBER-3 findings that adding a CDK4/6i may also confer benefit in this patient population, although we were not powered to identify differences in ET-backbones. In our study, the cohort of 167 patients with ESR1m on CDK4/6i plus ET had a median PFS of 5.3 months—comparable to our overall PFS analysis. The group of 33 patients carrying such mutations and treated with ET alone had a median PFS of 3.1 months. A significant difference of 1.8 months between the two groups was seen in RMST analysis, but we highlight the limited number of patients in the ET alone arm.

Mutations in PIK3CA are also implicated in treatment resistance following CDK4/6i plus ET. However, these mutations drive signaling through estrogen-independent pathways (e.g., PI3K/AKT/mTOR) and provide the rationale for targeted therapies, such as alpelisib. The latter was evaluated in the post-CDK4/6i setting in the phase II BYlieve study, in which patients with PIK3CA alterations achieved a median PFS of 7.3 months [38]. The phase III CAPItello-291 trial has placed capivasertib (a pan-AKT inhibitor) plus fulvestrant as one of the most used strategies for patients with AKT pathway–altered disease following progression on ET, typically with CDK4/6i (HR for PFS, 0.50, 95% CI 0.38–0.65, *P* < 0.001 for combined treatment *versus* fulvestrant monotherapy) [39].

Moreover, emerging data from MAINTAIN and PACE trials suggest that rechallenge with CDK4/6i plus ET may be an alternative for patients with PIK3CA mutations [13, 14]. In our meta-analysis, 98 patients harbored such an alteration and received CDK4/6i plus ET. They achieved a median PFS of 4.7 months, consistent with the 5.8-month PFS seen in our overall cohort. Our findings align with previous studies. Nonetheless, there is still no randomized study comparing the different treatments and therefore no agreed-upon strategy for patients with PIK3CA mutations progressing after CDK4/6i plus ET. Current decisions are based on multiple factors, including eligibility for targeted therapies, duration of benefit on first CDK4/6i, and presence of co-mutations. More evidence is needed to further establish the optimal regimen for this patient population.

Molecular drivers of resistance and their effect on subsequent lines of therapy remain to be elucidated. The randomized phase III PADA-1 trial provided an interesting insight into early detection of resistance-associated mutations [40]. Patients were randomized to continue first-line palbociclib plus AI or switch to palbociclib plus fulvestrant upon rise of ESR1 alterations on circulating tumor DNA and no radiographic evidence of disease progression. Patients who switched regimens had a remarkable 39% improvement in PFS. These findings suggest that endocrine resistance may precede CDK4/6i resistance [29]. It may also indicate that molecular progression can be earlier detected than clinical progression, although more data are still needed to establish biomarker-driven treatment strategies.

In this meta-analysis, only two of the studies included CDK4/6i plus ET combined with other therapies. In our sensitivity analysis removing them, a similar PFS was observed. However, data suggest that CDK4/6i could have a synergistic activity with other targeted therapies and provide additional benefit [40, 41]. Pre-clinical studies showed that CDK4/6i may trigger antitumor immunity [42]. Interestingly, the PACE trial failed to show superiority of palbociclib plus fulvestrant over fulvestrant alone, but the addition of the anti-PDL1 inhibitor avelumab to the combined regimen conferred longer PFS. Concomitant inhibition of other pathways may also enhance efficacy [14]. The early phase TRINITI-1 studied the association of the mTOR inhibitor everolimus with ribociclib plus exemestane and reported a CBR of 41.1%—meeting its primary endpoint [31].

Our findings support the benefit of CDK4/6i plus ET rechallenge over ET alone in patients with HR-positive HER2-negative advanced/metastatic BC following progression on initial CDK4/6i plus ET. Nonetheless, several unanswered questions are yet to be addressed. Firstly, whether switching to another CDK4/6i upon rechallenge is indeed superior to retreatment with the same agent remains unclear. Although abemaciclib was associated with longer PFS in this study, the optimal choice of CDK4/6i and ET partners also warrants investigation. Combining CDK4/6i plus ET with targeted therapies may add benefit and further broaden the treatment landscape of this patient population. Understanding resistance mechanisms and the role of biomarker-guided approaches is yet another challenge. Ongoing studies may address some of these challenges (NCT04546009, NCT04862663, NCT05563220, NCT05386108, NCT04964934).

This study has limitations. This was a reconstructed rather than true individual-level data meta-analysis and we relied on published Kaplan–Meier curves to perform analyses. Thus, it is subject to reconstruction errors and availability of data. Important subgroup analyses (i.e., optimal ET partner) and the impact of certain baseline factors on survival rates could not be explored. Even when using robust Kaplan–Meier reconstructed individual-level data methods, some subgroup analyses remain only exploratory and should be interpreted cautiously as differences in patient population and setting precluded us from performing reliable head-to-head comparisons (i.e., optimal CDK4/6i agent in the rechallenge setting). Confounding factors were acknowledged but could not be adjusted due to the lack of true individual patient data, thereby hindering our understanding of different treatment strategies: is switching CDK4/6i indeed superior to maintaining the same agent or were these findings influenced by the predominant use of abemaciclib in the rechallenge setting? Moreover, OS data was mostly immature; therefore, it could not be truly assessed. Different study designs, lines of therapy, and eligibility criteria from studies add heterogeneity and may affect the interpretability of data. For some subgroups, only a limited number of patients were included.

## Conclusions

This meta-analysis of reconstructed time-to-event data including 1396 patients with HR-positive, HER2-negative advanced or metastatic breast cancer suggests that rechallenge with CDK4/6 inhibitors plus ET may represent a potential post-progression treatment strategy compared with ET alone. In our analysis, CDK4/6i rechallenge was associated with a 29% reduction in the risk of progression or death, further confirmed by analysis restricted to randomized studies. Patients with ESR1 and PIK3CA mutations also derived PFS benefit from this strategy. Among the three agents used in the rechallenge setting, abemaciclib was associated with the longest PFS. Unanswered questions remain as to the optimal CDK4/6i agent and ET partner and whether switching or not to a different CDK4/6i upon rechallenge affects outcomes. Further prospective studies are needed to better define the optimal rechallenge strategy and the role of biomarker-guided treatment selection.

## Supplementary Information

Supplementary Material 1

## Data Availability

Data used in this study is available upon reasonable request to the corresponding author.

## Abbreviations

AI: Aromatase inhibitor
AKT: Protein kinase B
BC: Breast cancer
CBR: Clinical benefit rate
CDK: Cyclin-dependent kinase
CDK4/6i: Cyclin-dependent kinase 4/6 inhibitor
CI: Confidence interval
CT: Clinical trial
DNA: Deoxyribonucleic acid
EMA: European medicines agency
ER: Estrogen receptor
ERBB2: Erb-b2 receptor tyrosine kinase 2
ESMO: European society for medical oncology
ESR1: Estrogen receptor 1 gene
ESR1m: Estrogen receptor 1 gene mutation
ET: Endocrine therapy
FDA: U.S. food and drug administration
HER2: Human epidermal growth factor receptor 2
HR: Hazard ratio
IPD: Individual patient data
mTOR: Mammalian target of rapamycin
OR: Odds ratio
ORR: Objective response rate
OS: Overall survival
PD-L1: Programmed death-ligand 1
PFS: Progression-free survival
PH: Proportional hazards
PI3K: Phosphatidylinositol 3-kinase
PIK3CA: Phosphatidylinositol-4,5-bisphosphate 3-kinase catalytic subunit alpha
PRISMA: Preferred reporting items for systematic reviews and meta-analyses
PROSPERO: International prospective register of systematic reviews
RMST: Restricted mean survival time
RoB 2: Cochrane risk of bias tool for randomized trials
ROBINS-I: Risk of bias in non-randomized studies of interventions
SERD: Selective estrogen receptor degrader
SERM: Selective estrogen receptor modulator
