# Supplementary material for: CDK4/6 inhibitors rechallenge post-progression in HR-positive HER2-negative advanced/metastatic breast cancer patients: a meta-analysis of Kaplan–Meier-reconstructed individual-level data
Source: Breast Cancer Res. 2026 May 27;28:100. doi: 10.1186/s13058-026-02311-x (PMC13214152; doi:10.1186/s13058-026-02311-x)

Supplementary Material

CDK4/6 Inhibitors Rechallenge Post-Progression in HR-positive HER2-negative Advanced/Metastatic Breast Cancer Patients: A Meta-Analysis of Kaplan-Meier-Reconstructed Individual-Level Data

Table of Contents

Table S1: Preferred Reporting Items for Systematic Reviews and Meta-Analysis (PRISMA) Checklist for the Manuscript (A), and for the Abstract (B)......2

(A) Manuscript Prisma Checklist......2

(B) Abstract Prisma Checklist......5

Table S2: Full search strategy used in each database......6

Table S3: List of studies excluded after full review......7

Table S4: Restricted mean survival time (RMST) for patients harboring ESR1 and PIK3CA alterations at 12 months......9

Table S5: Median survival and 12-month survival probability across groups......10

Table S6: Grambsch-Therneau test......10

Table S7: Sensitivity analyses......11

Table S8: Quality assessment for studies included in this systematic review and meta-analysis......12

(A) Risk of bias summary for non-randomized studies (ROBINS-I)......12

(B) Risk of bias summary for randomized studies (RoB 2)......12

Figures......13

Fig.S1: PFS in CDK4/6i+ET with (A) Different CDK4/6i and (B) Same CDK4/6i at rechallenge......13

Fig.S2: PFS considering the three CDK4/6i used in the rechallenge......14

Fig.S3: PFS according to CDK4/6i agent used in rechallenge......15

(A) Palbo+ET versus ET alone......15

(B) Ribo+ET versus ET alone......15

(C) Abema+ET versus ET alone......16

Fig.S4: PFS according to ET backbone in the rechallenge......17

Fig.S5: PFS across different subgroups......18

Fig.S6: Response rate of the CDK4/6i+ET rechallenge group......19

(A) Objective response rate......19

(B) Clinical benefit rate......19

Fig.S7: Binary response outcomes......20

(A) Objective response rate......20

(B) Clinical benefit rate......20

Tables

Table S1: Preferred Reporting Items for Systematic Reviews and Meta-Analysis (PRISMA) Checklist for the Manuscript (A), and for the Abstract (B).

(A) Manuscript Prisma Checklist

Section and Topic	Item #	Checklist item	Location where item is reported
**TITLE**			
**Title**	**1**	Identify the report as a systematic review.	**Page 1**
**ABSTRACT**			
**Abstract**	**2**	See the PRISMA 2020 for Abstracts checklist.	**Table S1B**
**INTRODUCTION**			
**Rationale**	**3**	Describe the rationale for the review in the context of existing knowledge.	**Page 4**
**Objectives**	**4**	Provide an explicit statement of the objective(s) or question(s) the review addresses.	**Page 4**
**METHODS**			
**Eligibility criteria**	**5**	Specify the inclusion and exclusion criteria for the review and how studies were grouped for the syntheses.	**Page 5**
**Information sources**	**6**	Specify all databases, registers, websites, organizations, reference lists and other sources searched or consulted to identify studies. Specify the date when each source was last searched or consulted.	**Page 5**
**Search strategy**	**7**	Present the full search strategies for all databases, registers and websites, including any filters and limits used.	**Table S2**
**Selection process**	**8**	Specify the methods used to decide whether a study met the inclusion criteria of the review, including how many reviewers screened each record and each report retrieved, whether they worked independently, and if applicable, details of automation tools used in the process.	**Page 5**
**Data collection process**	**9**	Specify the methods used to collect data from reports, including how many reviewers collected data from each report, whether they worked independently, any processes for obtaining or confirming data from study investigators, and if applicable, details of automation tools used in the process.	**Page 5**
**Data items**	**10a**	List and define all outcomes for which data were sought. Specify whether all results that were compatible with each outcome domain in each study were sought (e.g. for all measures, time points, analyses), and if not, the methods used to decide which results to collect.	**Pages 5, 6**
	**10b**	List and define all other variables for which data were sought (e.g. participant and intervention characteristics, funding sources). Describe any assumptions made about any missing or unclear information.	**Pages 5, 6**

**Study risk of bias assessment**	**11**	Specify the methods used to assess risk of bias in the included studies, including details of the tool(s) used, how many reviewers assessed each study and whether they worked independently, and if applicable, details of automation tools used in the process.	**Page 5**
**Effect measures**	**12**	Specify for each outcome the effect measure(s) (e.g. risk ratio, mean difference) used in the synthesis or presentation of results.	**Page 5**
**Synthesis methods**	**13a**	Describe the processes used to decide which studies were eligible for each synthesis (e.g. tabulating the study intervention characteristics and comparing against the planned groups for each synthesis (item #5)).	**Pages 6, 7**
	**13b**	Describe any methods required to prepare the data for presentation or synthesis, such as handling of missing summary statistics, or data conversions.	**Pages 6, 7**
	**13c**	Describe any methods used to tabulate or visually display results of individual studies and syntheses.	**Pages 6, 7**
	**13d**	Describe any methods used to synthesize results and provide a rationale for the choice(s). If meta-analysis was performed, describe the model(s), method(s) to identify the presence and extent of statistical heterogeneity, and software package(s) used.	**Pages 6, 7**
	**13e**	Describe any methods used to explore possible causes of heterogeneity among study results (e.g. subgroup analysis, meta-regression).	**Page 6**
	**13f**	Describe any sensitivity analyses conducted to assess robustness of the synthesized results.	**Page 6**
**Reporting bias assessment**	**14**	Describe any methods used to assess risk of bias due to missing results in a synthesis (arising from reporting biases).	**NA**
**Certainty assessment**	**15**	Describe any methods used to assess certainty (or confidence) in the body of evidence for an outcome.	**NA**
RESULTS			
**Study selection**	**16a**	Describe the results of the search and selection process, from the number of records identified in the search to the number of studies included in the review, ideally using a flow diagram.	**Page 7**
	**16b**	Cite studies that might appear to meet the inclusion criteria, but which were excluded, and explain why they were excluded.	**Table S3**
**Study characteristics**	**17**	Cite each included study and present its characteristics.	**Table 1**
**Risk of bias in studies**	**18**	Present assessments of risk of bias for each included study.	**Table S4**

**Results of individual studies**	**19**	For all outcomes, present, for each study: (a) summary statistics for each group (where appropriate) and (b) an effect estimate and its precision (e.g. confidence/credible interval), ideally using structured tables or plots.	**Figures 2-4**
**Results of syntheses**	**20a**	For each synthesis, briefly summarise the characteristics and risk of bias among contributing studies.	**Table S4**
	**20b**	Present results of all statistical syntheses conducted. If meta-analysis was done, present for each the summary estimate and its precision (e.g. confidence/credible interval) and measures of statistical heterogeneity. If comparing groups, describe the direction of the effect.	**Pages 7, 8**
	**20c**	Present results of all investigations of possible causes of heterogeneity among study results.	**Page 9**
	**20d**	Present results of all sensitivity analyses conducted to assess the robustness of the synthesized results.	**Page 9**
**Reporting biases**	**21**	Present assessments of risk of bias due to missing results (arising from reporting biases) for each synthesis assessed.	**NA**
**Certainty of evidence**	**22**	Present assessments of certainty (or confidence) in the body of evidence for each outcome assessed.	**NA**
DISCUSSION			
**Discussion**	**23a**	Provide a general interpretation of the results in the context of other evidence.	**Page 9**
	**23b**	Discuss any limitations of the evidence included in the review.	**Page 12**
	**23c**	Discuss any limitations of the review processes used.	**Page 12**
	**23d**	Discuss implications of the results for practice, policy, and future research.	**Page 12**
OTHER INFORMATION			
**Registration and protocol**	**24a**	Provide registration information for the review, including register name and registration number, or state that the review was not registered.	**Page 5**
	**24b**	Indicate where the review protocol can be accessed, or state that a protocol was not prepared.	**Page 5**
	**24c**	Describe and explain any amendments to information provided at registration or in the protocol.	**NA**
**Support**	**25**	Describe sources of financial or non-financial support for the review, and the role of the funders or sponsors in the review.	**Page 13**
**Competing interests**	**26**	Declare any competing interests of review authors.	**Page 13**

**Availability of data, code and other materials**	**27**	Report which of the following are publicly available and where they can be found: template data collection forms; data extracted from included studies; data used for all analyses; analytic code; any other materials used in the review.	**Page 13**

NA: not available

(B) Abstract Prisma Checklist

Section and Topic	Item #	Checklist item	Reported (Yes/No)
**TITLE**			
**Title**	1	Identify the report as a systematic review.	**Yes**
BACKGROUND			
**Objectives**	2	Provide an explicit statement of the main objective(s) or question(s) the review addresses.	**Yes**
METHODS			
**Eligibility criteria**	3	Specify the inclusion and exclusion criteria for the review.	**Yes**
**Information sources**	4	Specify the information sources (e.g. databases, registers) used to identify studies and the date when each was last searched.	**Yes**
**Risk of bias**	5	Specify the methods used to assess risk of bias in the included studies.	**No**
**Synthesis of results**	6	Specify the methods used to present and synthesise results.	**Yes**
RESULTS			
**Included studies**	7	Give the total number of included studies and participants and summarise relevant characteristics of studies.	**Yes**
**Synthesis of results**	8	Present results for main outcomes, preferably indicating the number of included studies and participants for each. If meta-analysis was done, report the summary estimate and confidence/credible interval. If comparing groups, indicate the direction of the effect (i.e. which group is favoured).	**Yes**
DISCUSSION			
**Limitations of evidence**	9	Provide a brief summary of the limitations of the evidence included in the review (e.g. study risk of bias, inconsistency and imprecision).	**No**
**Interpretation**	10	Provide a general interpretation of the results and important implications.	**Yes**
OTHER			
**Funding**	11	Specify the primary source of funding for the review.	**No**
**Registration**	12	Provide the register name and registration number.	**No**

Table S2: Full search strategy used in each database.

Database
**PubMed**
(Breast Neoplasms[mh]) AND ("human epidermal growth factor receptor 2 negative" OR "HER2-negative" OR "HER2-zero" OR "HER2 negative" OR "HER2-" OR "ERBB2-negative" OR "ERBB2 negative") AND ((Cyclin-Dependent Kinase Inhibitor Proteins[mh]) OR “CDK4/6i” OR "CDK4/6 inhibitors" OR palbociclib OR ribociclib OR abemaciclib) AND (Retreatment[MeSH] OR rechallenge OR "re-treatment" OR retreatment OR progression OR resume OR relapse OR recurrence OR "re-introducing" OR "re-introduction" OR "re-initiation" OR subsequent OR progression)
**Embase**
('breast cancer':ab,ti) AND ('her2-negative':ab,ti OR 'her2 zero':ab,ti OR 'her2 negative':ab,ti OR 'her2-':ab,ti OR 'erbb2-negative':ab,ti OR 'erbb2 negative':ab,ti) AND ('cyclin dependent kinase inhibitor':ab,ti OR 'cdk4 6 inhibitor':ab,ti OR 'cdk4/6 inhibitor':ab,ti OR palbociclib:ab,ti OR ribociclib:ab,ti OR abemaciclib:ab,ti) AND ('retreatment':ab,ti OR rechallenge:ab,ti OR 're-treatment':ab,ti OR retreatment:ab,ti OR progression:ab,ti OR resume:ab,ti OR relapse:ab,ti OR recurrence:ab,ti OR 're-introducing':ab,ti OR 're-introduction':ab,ti OR 're-initiation':ab,ti)
**Cochrane**
("breast cancer" OR "breast neoplasms" OR "breast tumor" OR "breast tumour" OR "breast carcinoma" OR "mammary carcinoma" OR "mammary neoplasms") AND ("human epidermal growth factor receptor 2 negative" OR "HER2-negative" OR "HER2-zero" OR "HER2 negative" OR "HER2-" OR "ERBB2-negative" OR "ERBB2 negative") AND ("Cyclin-Dependent Kinase Inhibitor Proteins" OR CDK4/6i OR "CDK4/6 inhibitors" OR palbociclib OR ribociclib OR abemaciclib) AND (retreatment OR "re-treatment" OR rechallenge OR progression OR resume OR relapse OR recurrence OR "re-introducing" OR "re-introduction" OR "re-initiation")

Table S3: List of studies excluded after full review.

Author	Study Title	Exclusion criteria
Bardia et al., 2022	AMEERA-1 phase 1/2 study of amcenestrant, SAR439859, in postmenopausal women with ER-positive/HER2-negative advanced breast cancer	Phase I
Brett et al., 2023	A Gene Panel Associated With Abemaciclib Utility in ESR1-Mutated Breast Cancer After Prior Cyclin-Dependent Kinase 4/6-Inhibitor Progression	Real-world
Chandarlapaty et al., 2022	Updated data from AMEERA-1: Phase 1/2 study of amcenestrant (SAR439859), an oral selective estrogen receptor (ER) degrader (SERD), combined with palbociclib in postmenopausal women with ER+/HER2- advanced breast cancer	Phase I
de Luna Aguilar et al., 2023	Clinical Experience with Abemaciclib in Patients Previously Treated with Another CDK 4/6 Inhibitor in a Tertiary Hospital: A Case Series Study	Real-world
Delaloge et al., 2022	First line aromatase inhibitor (AI) + palbociclib with randomized switch to fulvestrant + palbociclib upon detection of circulating ESR1 mutation in HR+ HER2-metastatic breast cancer patients: Global safety results of PADA-1, a UCBG-GINECO phase III trial	Lack of CDK4/6i rechallenge
Dos Anjos et al., 2019	A large retrospective analysis of CDK 4/6 inhibitor retreatment in ER+ metastatic breast cancer (MBC)	Real-world
ELAINE 2, 2024	Baseline genomic alterations and the activity of lasofoxifene (LAS) plus abemaciclib (Abema) in patients with ER+/HER2-metastatic breast cancer (mBC): the ELAINE 2 study	Another publication already included
EUCTR2021-002301-10-ES	Abemaciclib plus Fulvestrant compared to Placebo plus Fulvestrant in HR+, HER2-, Advanced or Metastatic Breast Cancer previously treated with a CDK4/6 Inhibitor and Endocrine Therapy	Another publication already included
Eziokwu et al, 2020	Real-World outcomes of cyclin-dependent kinase inhibitors continued beyond first disease progression in hormone receptor-positive metastatic breast cancer	Real-world
Giordano et al., 2024	International phase 3 clinical trial evaluating PF-07220060 plus fulvestrant in patients with HR+/HER22 advanced/metastatic breast cancer with progression after a prior CDK4/6 inhibitor	Protocol only
Goetz et al., 2025	ELAINE 3: phase 3 study of lasofoxifene plus abemaciclib to treat ER+/HER2-, ESR1-mutated, metastatic breast cancer	Protocol only
Hurvitz et al., 2022	Ribociclib, everolimus, exemestane triplet therapy in HR+/HER2-advanced breast cancer after progression on a CDK4/6 inhibitor: Final efficacy, safety, and biomarker results from TRINITI-1	Abstract
Jiang et al., 2023	Abemaciclib plus endocrine therapy versus chemotherapy after progression on prior palbociclib in HR+/HER2metastatic breast cancer: A single center real- world study in China	Real-world
Kruse et al., 2023	Treatment patterns and outcomes associated with sequential and non-sequential use of CDK4 & 6 inhibitors in patients with HR+, HER2− MBC in the real world	Real-world
Layman et al., 2024	Gedatolisib in combination with palbociclib and endocrine therapy in women with hormone receptor-positive, HER2negative advanced breast cancer: results from the dose expansion groups of an open-label, phase 1b study	Phase I
Lim et al., 2022	Phase 3 ENABLAR-2 study to evaluate enobosarm and abemaciclib combination compared to estrogen-blocking agent for the second-line	Protocol only

	treatment of AR+, ER+, HER2- metastatic breast cancer in patients who previously received palbociclib and estrogen-blocking agent combination therapy.	
Mai et al., 2024	Predictors of response to CDK4/6i retrial after prior CDK4/6i failure in ER+ metastatic breast cancer	Real-world
Mainor et al., 2023	A phase I trial of palbociclib (palbo) and bosutinib (bos) with fulvestrant (fulv) in patients (pts) with hormone receptor-positive, HER2-negative (HR+/HER2-) metastatic breast cancer (MBC) refractory to an aromatase inhibitor (AI) and a CDK4/6 inhibitor (CDK4/6i)	Phase I
Martin et al, 2022	Systemic Therapies Following Progression on First-line CDK4/6-inhibitor Treatment: Analysis of Real-world Data	Real-world
Nishimura et al., 2022	Clinical evaluation of the efficacy and liquid molecular analysis of abemaciclib rechallenge upon progression to abemaciclib combination therapies for ER-positive HER2-negative metastatic breast cancer patients	Protocol only
PADA 1, 2022	Switch to fulvestrant and palbociclib versus no switch in advanced breast cancer with rising ESR1 mutation during aromatase inhibitor and palbociclib therapy (PADA-1): a randomised, open-label, multicentre, phase 3 trial	Lack of rechallenge
Rinn et al., 2024	Design of Active Phase 3 ENABLAR-2 Study Evaluating Enobosarm +/- Abemaciclib in Patients with AR+ER+HER2-2nd-Line Metastatic Breast Cancer Following Tumor Progression on an Estrogen Blocking Agent Plus Palbociclib or Ribociclib	Protocol only
Roy et al., 2023	A phase I trial of palbociclib and bosutinib with fulvestrant in patients with metastatic hormone receptor positive and HER2 negative (HR+ HER2-) breast cancer refractory to an aromatase inhibitor	Phase I
Seki et la, 2022	Subsequent-abemaciclib Treatment After Disease Progression on Palbociclib in Patients With ER-positive HER2-negative Metastatic Breast Cancer	Wrong study design
Tao et al., 2023	Phase II trial of palbociclib with fulvestrant in individuals with hormone receptor-positive, HER2negative metastatic breast cancer with disease progression after palbociclib with an aromatase inhibitor	Abstract
Wander et al., 2020	Clinical Outcomes With Abemaciclib After Prior CDK4/6 Inhibitor Progression in Breast Cancer: A Multicenter Experience	Real-world
Wesolowski et al., 2023	PD13-05 Updated results of a Phase 1b study of gedatolisib plus palbociclib and endocrine therapy in women with hormone receptor positive advanced breast cancer: Subgroup analysis by PIK3CA mutation status	Phase I/abstract
West et al., 2023	Real-World Evaluation of Disease Progression After CDK 4/6 Inhibitor Therapy in Patients with Hormone Receptor-Positive Metastatic Breast Cancer	Real-world
Yuan et al., 2023	Efficacy and safety of abemaciclib-based therapy versus tucidinostat-based therapy after progression on palbociclib in patients with HR+HER2− metastatic breast cancer	Real-world

Table S4: Restricted mean survival time (RMST) for patients harboring ESR1 and PIK3CA alterations at 12 months.

	RMST in months (95% CI)			
	**CDK4/6i+ET**	**ET alone**	**Difference (95% CI)**	**P-value**
**ESR1m**	6.1 (5.5 – 6.8)	4.3 (3.1 – 5.6)	1.8 (0.4 – 3.2)	**P = 0.01**
**PIK3CAm**	5.9 (5.0–6.7)	5.3 (3.3–7.2)	0.6 (−1.6 – 2.7)	P = 0.60

Table S5: Median survival and 12-month survival probability across groups.

Analysis	Group	Median survival (95% CI), months	12-month survival probability (95% CI)*
PFS	CDK4/6i+ET	5.8 (5.5-7.1)	26.2% (22.9-29.9%)
	ET alone	3.7 (3.6-4.8)	16.1% (12.9-20.3%)
OS	CDK4/6i+ET	NA	80.6% (76-85.6%)
	ET alone	NA	80.4% (72.9-88.8%)
PFS - different CDK4/6i in rechallenge	CDK4/6i+ET	7.33 (5.9-8.4)	32.9% (28.5-38.1%)
	ET alone	3.8 (3.6-5.4)	17.1% (13.3-21.9%)
PFS - same CDK4/6i in earlier lines and rechallenge	CDK4/6i+ET	4.6 (3.7-5.6)	16.8% (12.7-22.2%)
	ET alone	3.6 (3.0-5.4)	13.5% (8-23.3%)
PFS in palbo+ET vs ET alone	palbo+ET	4.6 (3.7-5.7)	16.3% (12.3-21.7%)
	ET alone	3.6 (3.2-5.5)	14.7% (8.6-25.3%)
PFS in ribo+ET vs ET alone	ribo+ET	5.5 (4.2-7.5)	30.4% (23.2-39.8%)
	ET alone	2.9 (2.6-5.0)	7.3% (2.8-18.6%)
PFS in abema+ET vs ET alone	abema+ET	7.9 (6.5-9.3)	33.9% (28.6-40.2%)
	ET alone	4.0 (3.7-5.5)	18.8% (14.6-24.2%)
PFS by ET	SERM	14.0 (8.0-NA)	54.3% (36.5-80.8%)
	SERD	6.0 (5.6-7.6)	27.8% (23.7-32.6%)
	AI	5.8 (3.7-9.7)	33.8% (24.8-46.2%)
PFS in ESR1m	CDK4/6i+ET	5.3 (3.6-7.4)	42.3% (35.0-51.1%)†
	ET alone	3.1 (2.1-5.7)	23.4% (11.7-46.7%)†
PFS in PIK3CAm	CDK4/6i+ET	4.7 (3.6-6.7)	39.3% (30.1-51.3%)†
	ET alone	2.8 (2.7-NA)	34.2% (18.0-65.1%)†

*Refers to survival probability at 12 months unless indicated otherwise; † indicates survival probability at 6 months.

Table S6: Grambsch-Therneau test.

Analysis	Grambsch-Therneau test		
	Chi-square	df	P-value
PFS CDK4/6i+ET vs ET alone	0.08	1	0.78
OS CDK4/6i+ET vs ET alone	0.17	1	0.68
PFS - different CDK4/6i	0.01	1	0.91
PFS - same CDK4/6i	0.32	1	0.57
PFS in palbo+ET vs ET alone	0.40	1	0.53
PFS in ribo+ET vs ET alone	1.76	1	0.18
PFS in abema+ET vs ET alone	0.83	1	0.36

Table S7: Sensitivity analyses.

	HR (95% CI)	P-value
**PFS including only randomized studies**	0.72 (0.63–0.82)	<0.001
**OS including only randomized studies**	0.94 (0.66–1.35)	0.75
**PFS leaving out studies with CDK4/6i+ET combined with other targeted therapies**	0.75 (0.65–0.85)	<0.001

Table S8: Quality assessment for studies included in this systematic review and meta-analysis.

(A) Risk of bias summary for non-randomized studies (ROBINS-I).

Study/ author	Bias due to confounding	Bias in selection of participants	Bias in classification of interventions	Bias due to deviations from intended interventions	Bias due to missing data	Bias in measurement of outcomes	Bias in selection of the reported result	Overall risk of bias judgment
**BioPER**	Moderate[a]	Low	Low	Low	Low	Low	Low	Moderate
**ELAINE 2**	Moderate[a]	Low	Low	Low	Low	Low	Low	Moderate
**TRINITI-1**	Moderate[a]	Low	Low	Low	Low	Low	Low	Moderate

a: all non-randomized studies are subject to bias due to confounding factors;

(B) Risk of bias summary for randomized studies (RoB 2).

Study	Bias from randomization process	Bias due to deviations from intended interventions	Bias due to missing outcome data	Bias in measurement of the outcomes	Bias in selection of the reported result	Overall risk of bias
**PACE**	Low	Low	Low	Some concerns[a]	Low	Some concerns
**PALMIRA**	Low	Low	Low	Some concerns[a]	Low	Some concerns
**postMONARCH**	Low	Low	Low	Low	Low	Low
**MAINTAIN**	Low	Low	Low	Low	Low	Low
**EMBER-3**	Low	Low	Low	Some concerns[a]	Low	Some concerns

a: these were not double-blinded randomized studies in which primary survival outcomes were investigator-assessed (only EMBER-3 provided data separately for blinded-independent central review and it was not available to all analyses); therefore, they are at risk of detection bias.

Figures

Fig.S1: PFS in CDK4/6i+ET with (A) Different CDK4/6i and (B) Same CDK4/6i at rechallenge.

(A) Different CDK4/6i at rechallenge

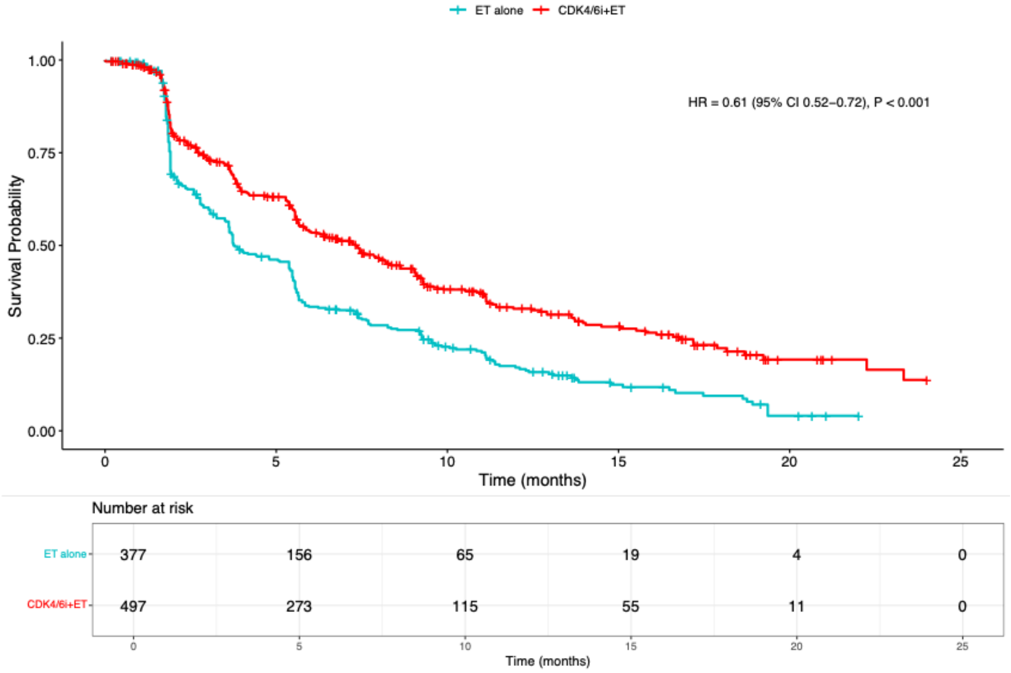

(B) Same CDK4/6i at rechallenge

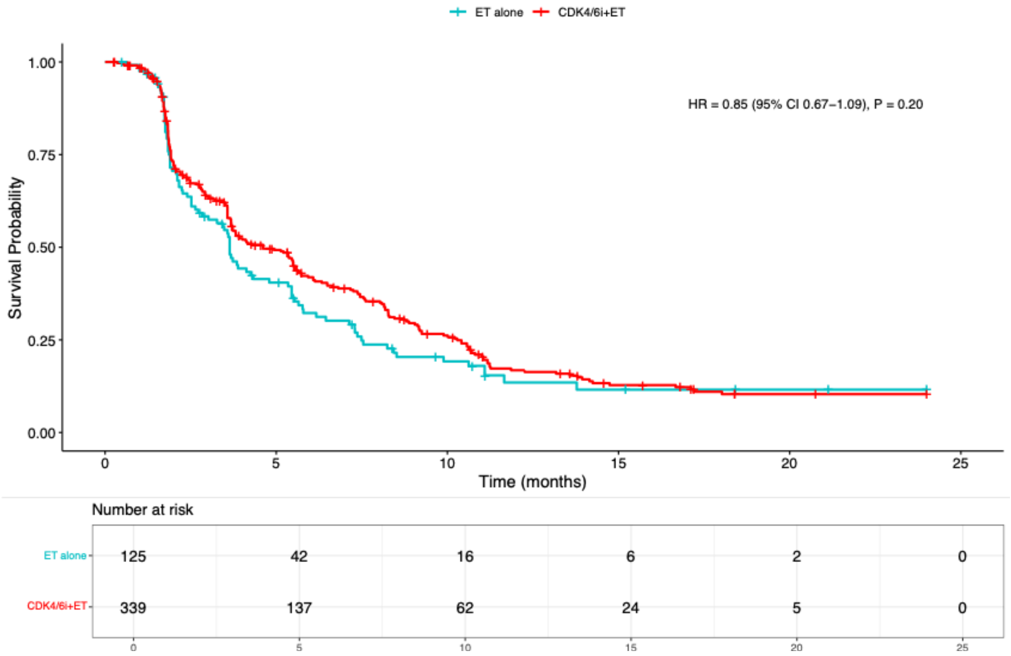

This analysis includes studies in which most patients received a different CDK4/6i at rechallenge: Post-MONARCH (59% of patients received palbociclib in the first line and were rechallenged with abemaciclib); EMBER-3 (64.7% of patients received palbociclib and were rechallenged with abemaciclib). In other studies included in this analysis (MAINTAIN, ELAINE 2, TRINITI 2), data was stratified accordingly or the majority (≈90%) of patients received a different CDK4/6i.

Fig.S2: PFS considering the three CDK4/6i used in the rechallenge.

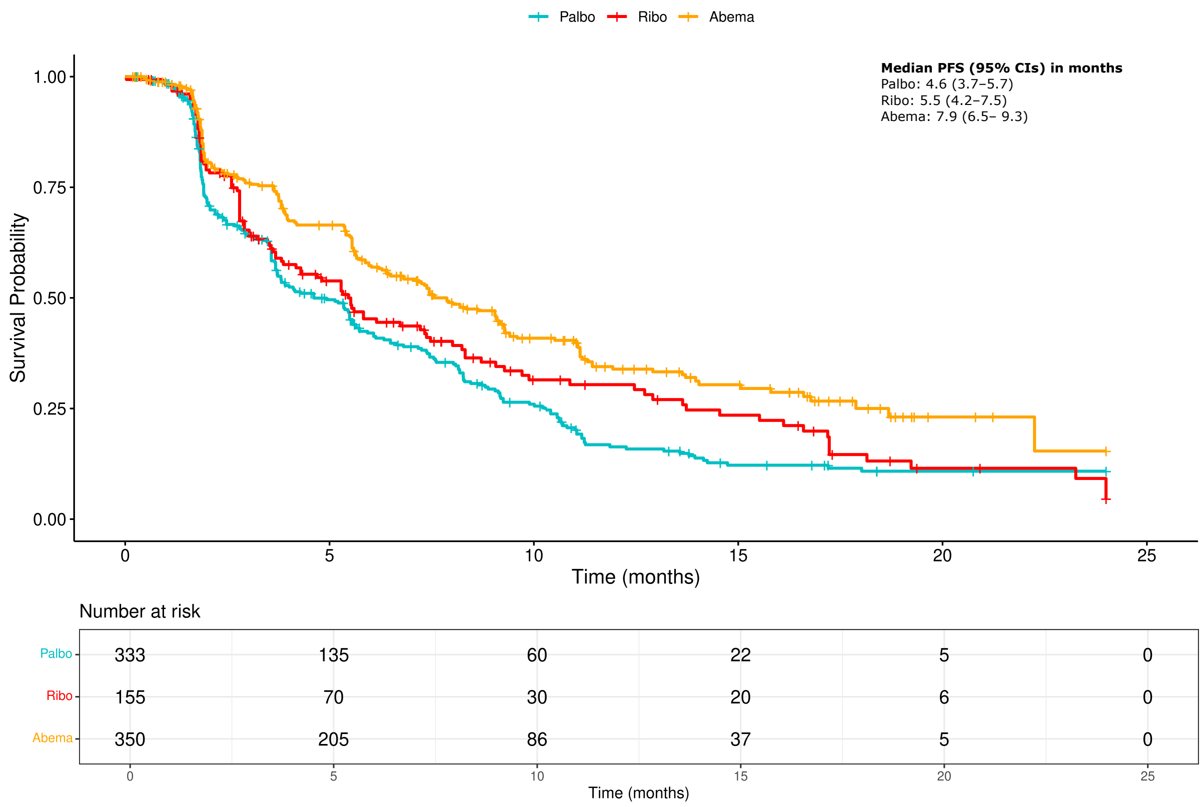

Fig.S3: PFS according to CDK4/6i agent used in rechallenge.

(A) Palbo+ET versus ET alone

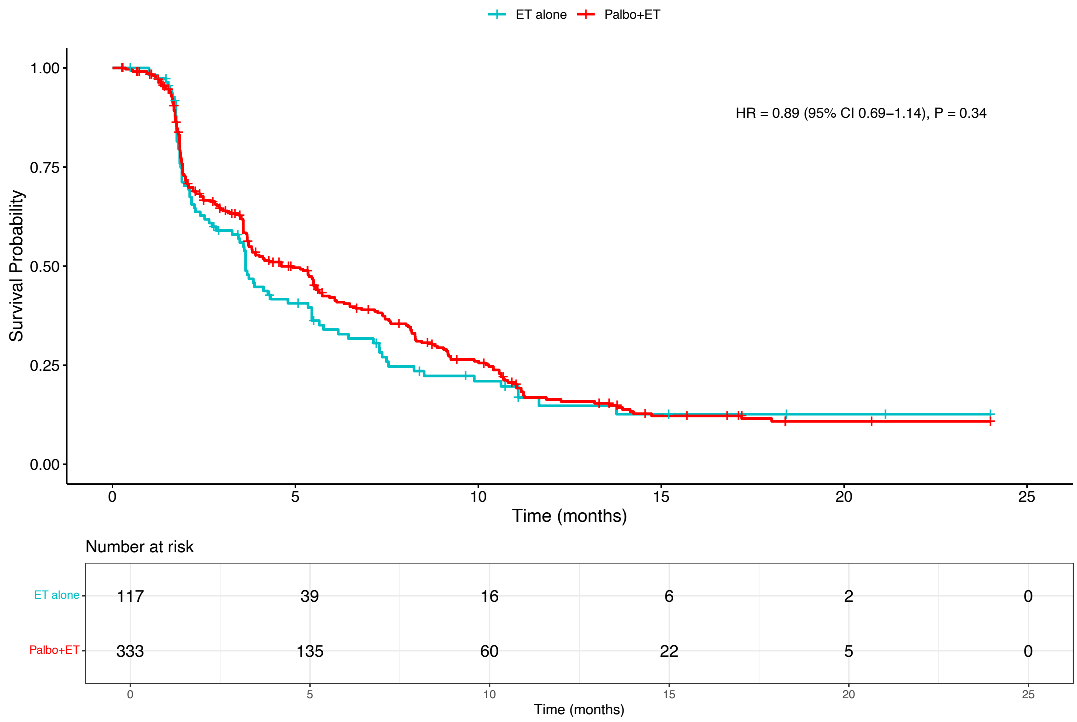

(B) Ribo+ET versus ET alone

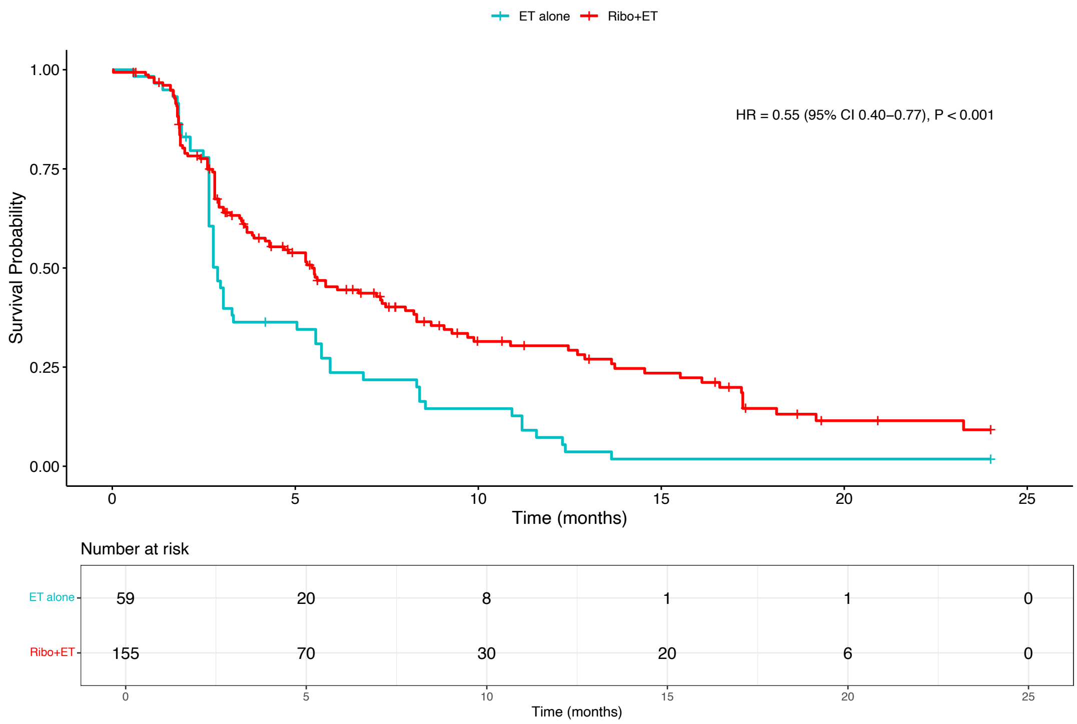

(C) Abema+ET versus ET alone

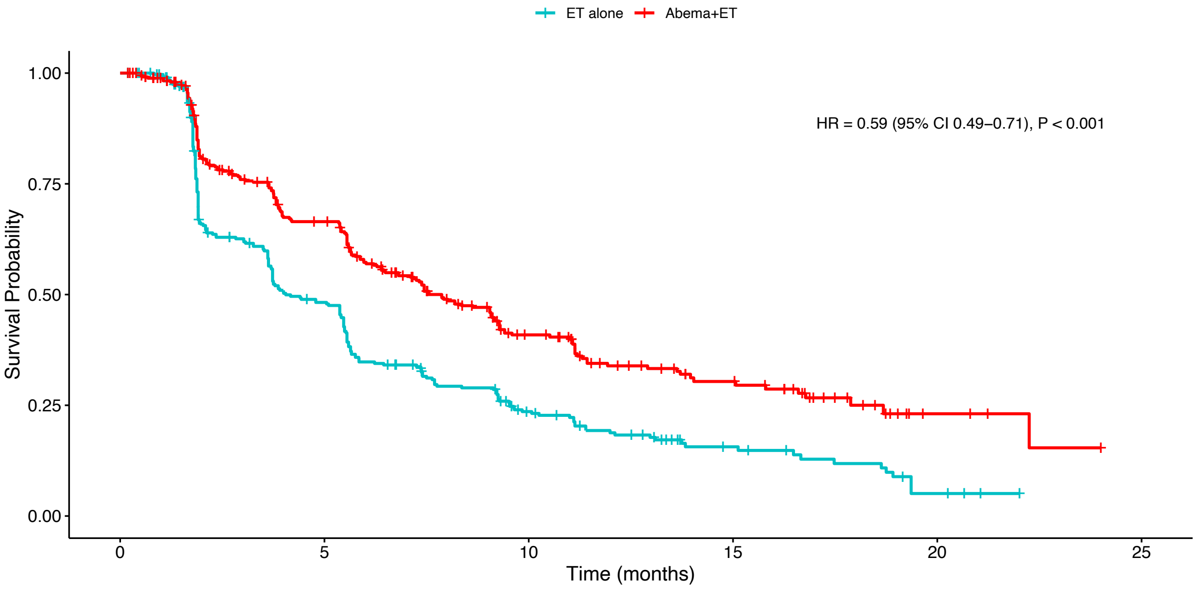

	0	5	10	15	20	25
ET alone	326	140	57	19	4	0
Abema+ET	350	205	86	37	5	0

Fig.S4: PFS according to ET backbone in the rechallenge.

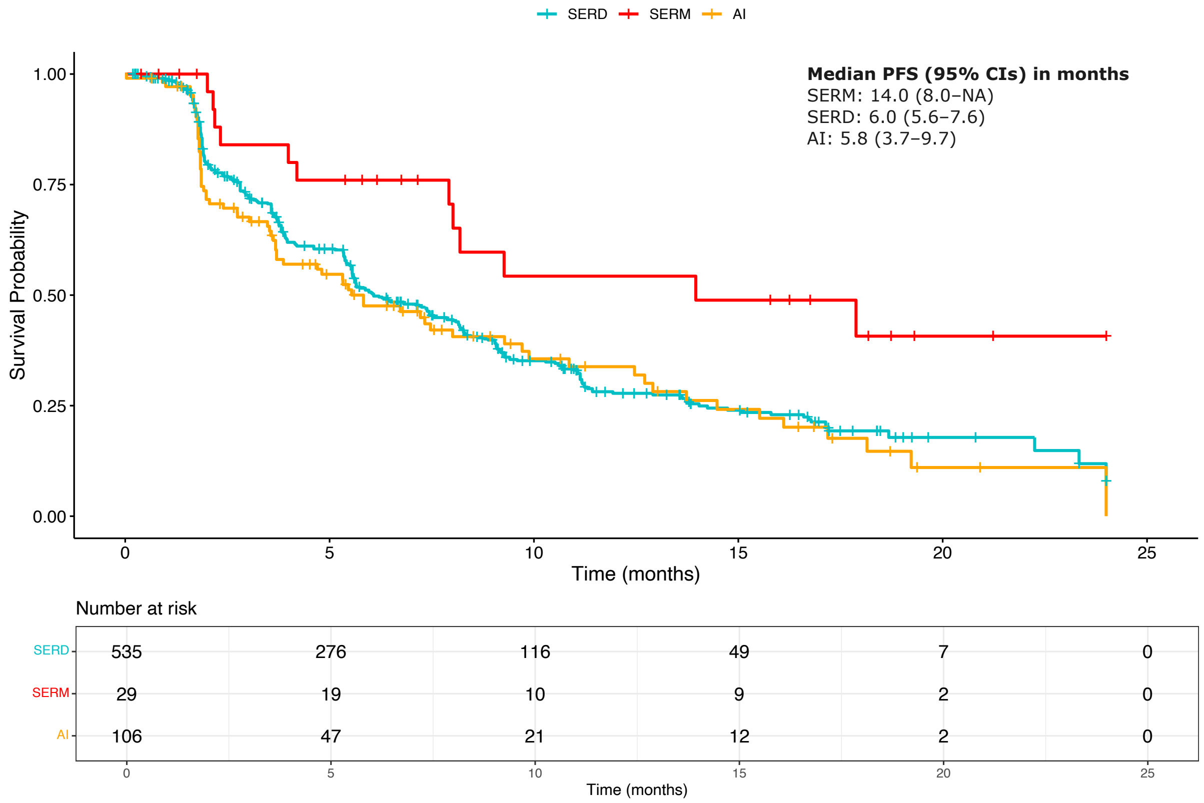

In this analysis, data was available for the following agents: SERM agents included lasofoxifene (n=29, used in ELAINE 2); SERD agents included fulvestrant (n=396, used in PACE, postMONARCH, MAINTAIN) and imlunestrant (n=139; used in EMBER-3); AI agents included exemestane (n=106, used in TRINITI-1 and MAINTAIN). Not all studies and agents could be included in this analysis due to lack of Kaplan-Meier PFS data stratified accordingly.

Fig.S5: PFS across different subgroups.

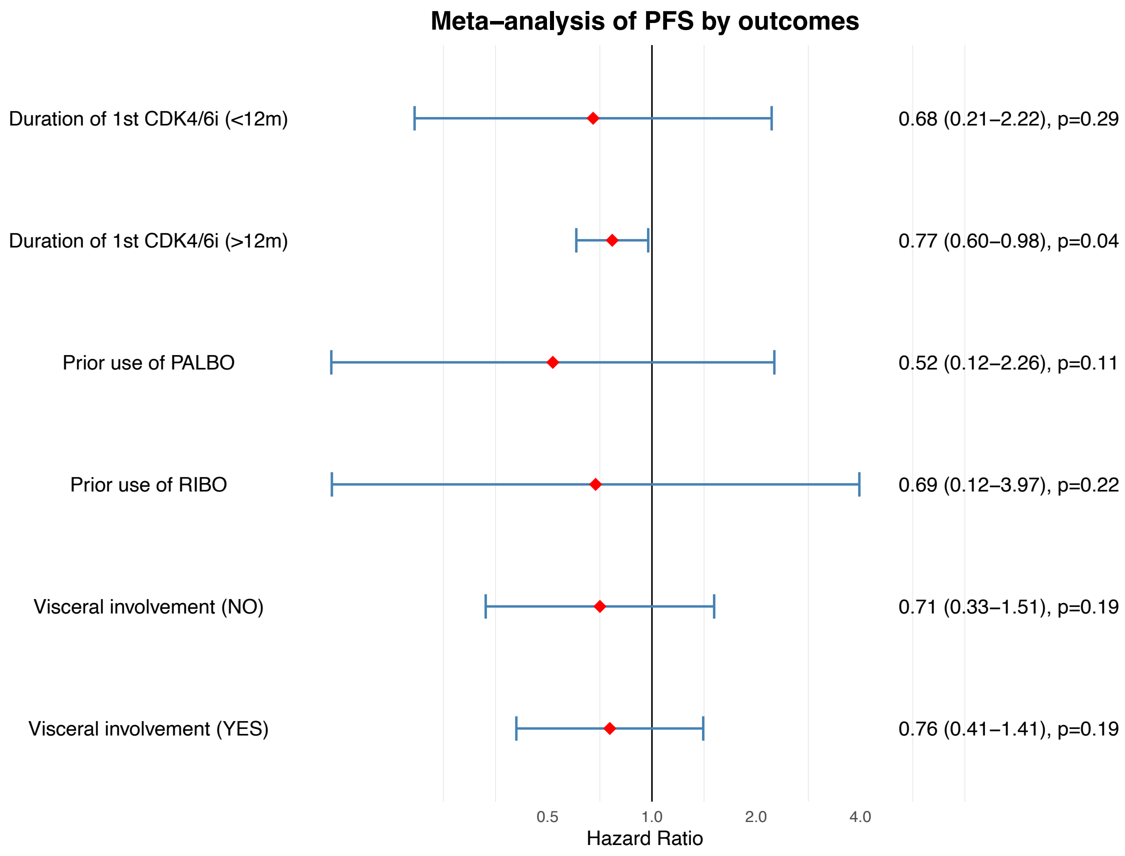

Fig.S6: Response rate of the CDK4/6i+ET rechallenge group.

(A) Objective response rate

Study	Events	Total	Proportion	95% C.I.
BioPER	2	32	6.25	[0.77; 20.81]
ELAINE 2	10	18	55.56	[30.76; 78.47]
MAINTAIN	7	35	20.00	[8.44; 36.94]
PACE	17	165	10.30	[6.12; 15.98]
PALMIRA	6	136	4.41	[1.64; 9.36]
post–MONARCH	36	182	19.78	[14.26; 26.32]
TRINITI–1	8	95	8.42	[3.71; 15.92]
**Total (95% CI)**	**86**	**663**	**13.33**	**[6.85; 24.35]**

Heterogeneity: $Tau^2 = 0.8277$; $Chi^2 = 38.96$, df = 6 ($P < 0.0001$); $I^2 = 84.6\%$

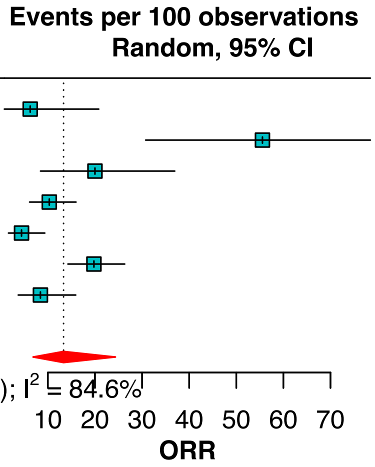

(B) Clinical benefit rate

Study	Events	Total	Proportion	95% C.I.
BioPER	11	32	34.38	[18.57; 53.19]
ELAINE 2	19	29	65.52	[45.67; 82.06]
MAINTAIN	21	49	42.86	[28.82; 57.79]
PACE	55	165	33.33	[26.20; 41.08]
PALMIRA	57	136	41.91	[33.51; 50.67]
post–MONARCH	50	182	27.47	[21.13; 34.57]
TRINITI–1	39	95	41.05	[31.06; 51.62]
**Total (95% CI)**	**252**	**688**	**38.89**	**[32.02; 46.23]**

Heterogeneity: $Tau^2 = 0.0982$; $Chi^2 = 19.83$, df = 6 ($P = 0.0030$); $I^2 = 69.7\%$

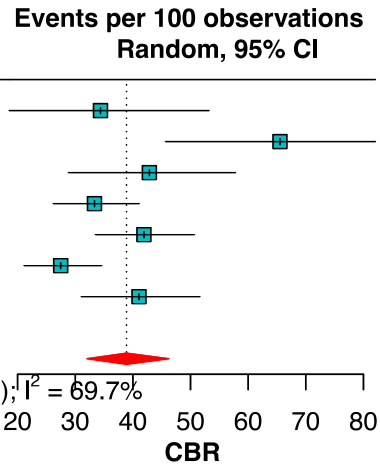

Fig.S7: Binary response outcomes

(A) Objective response rate

Studies	CDK4/6i+ET (N)	Total (N)	ET alone (N)	Total (N)	Weight	OR	CI
MAINTAIN	7	35	7	35	24.6%	1.00	[0.31; 3.23]
PACE	17	165	4	55	25.5%	1.46	[0.47; 4.56]
PALMIRA	6	136	1	62	10.3%	2.82	[0.33; 23.90]
post–MONARCH	36	182	11	186	39.6%	3.92	[1.93; 7.98]
**Total (95% CI)**					**100.0%**	**2.11**	**[0.99; 4.46]**

Heterogeneity: $Tau^2 = 0.2393$; $Chi^2 = 4.73$, df = 3 (P = 0.1923); $I^2 = 36.6\%$
Test for overall effect: Z = 1.94 (P = 0.052)

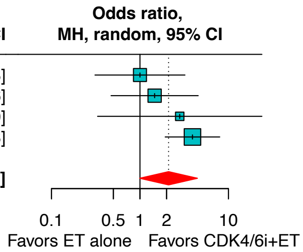

(B) Clinical benefit rate

Studies	CDK4/6i+ET (N)	Total (N)	ET alone (N)	Total (N)	Weight	OR	CI
MAINTAIN	21	49	14	57	16.9%	2.30	[1.01; 5.27]
PACE	55	165	16	55	24.4%	1.22	[0.63; 2.37]
PALMIRA	57	136	17	62	25.2%	1.91	[0.99; 3.67]
post–MONARCH	50	182	23	186	33.6%	2.68	[1.56; 4.63]
**Total (95% CI)**					**100.0%**	**1.98**	**[1.38; 2.85]**

Heterogeneity: $Tau^2 = 0.0248$; $Chi^2 = 3.38$, df = 3 (P = 0.3368); $I^2 = 11.2\%$
Test for overall effect: Z = 3.69 (P < 0.001)

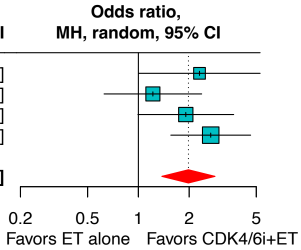

Supplement: Supplementary file 1 — Supplementary Material 1 [file 13058_2026_2311_MOESM1_ESM.pdf]
